# Is a Ratio Scale Assumption for Physician Ratings Justified? Comment on “What Patients Value in Physicians: Analyzing Drivers of Patient Satisfaction Using Physician-Rating Website Data”

**DOI:** 10.2196/18289

**Published:** 2020-10-26

**Authors:** Uwe Konerding

**Affiliations:** 1 Trimberg Research Academy University of Bamberg Bamberg Germany; 2 Department of Psychology and Psychotherapy Witten/Herdecke University Witten Germany

**Keywords:** patient satisfaction, modeling, method, scale level, measurement theory

In a paper published in the *Journal of Medical Internet Research*, Bidmon et al [1] investigated the relationships between 24 attributes of the service provided by physicians and 4 features of patient satisfaction with these physicians. The service attributes were assessed via 4-category rating scales coded from 1 for “strongly disagree” to 4 for “strongly agree” and the satisfaction features via 5-category rating scales from 1 for “bad” to 5 for “excellent.” The latter measures were rescaled to range from 1 to 4.

The authors stated that their measures “have to be assumed at ratio scale level.” Ratio scale level is a concept defined in the representational theory of measurement [2]. According to this theory, measurement means assigning numbers to objects so that the numerical relations reflect the empirical relations between objects. Examples of empirical relations are the relations that emerge when a double pan balance is used. When two objects *A* and *B* are compared, the relations “*A* is heavier than *B*,” “*B* is heavier than *A*,” or “*A* is as heavy as *B*” might emerge. When, for all objects, “*A* is heavier than *B*” and “*B* is heavier than *C*” imply that “*A* is heavier than *C*,” then these relations can be represented numerically by always assigning the higher number to the heavier object. This is the prototypical example of an ordinal scale. When two objects *A* and *B* are placed together on one pan of the scale and an individual object *C* on the other, empirical relations between pairs of objects and individual objects emerge that are analogous to those for comparing individual objects. When, for these relations, the same regularities hold as for the addition of numbers, these relations can be represented by assigning numbers so that number(*A*) + number(*B*) = number(*C*), if and only if *A* and *B* together are as heavy as *C*. Such an assignment is empirically determined except for the arbitrary choice of the measurement unit. The position of the zero-point is empirically determined. This is, by definition, a ratio scale.

In the measurements presented by Bidmon et al [1], the basic objects are statements regarding attributes or, respectively, physician practices. The relevant empirical relations are results of comparative judgments regarding these objects. Presently, there is no evidence that, for such judgments, empirical structures exist that permit the position of the zero-point to be determined. Hence, the zero-points for the rating scales analyzed by Bidmon et al [1] cannot be determined empirically. In fact, these zero-points are determined by arbitrary settings established by convention. Therefore, these scales are definitely not ratio scales.

In the absence of empirically determined zero-points, the analyses performed by Bidmon et al [1] are questionable. The authors estimated parameters for the model

ln(*Y*) = *b*_0_ + *b*_1_ ln(*X*)         (1)

where *Y* represents satisfaction with a specific feature and *X* is an agreement with a statement regarding a service attribute. The estimations for these parameters change when the locations of the zero-points are changed. Consequently, what these parameters tell us about the actual relationships between satisfaction and service attributes is unclear.

## References

[1] Bidmon S, Elshiewy O, Terlutter R, Boztug Y (2020). What Patients Value in Physicians: Analyzing Drivers of Patient Satisfaction Using Physician-Rating Website Data. J Med Internet Res.

[2] Krantz DH, Luce RD, Suppes P, Tversky A (1971). Foundations of Measurement: Additive and Polynomial Representations, Vol 1.

